# Tea Polyphenols and Their Preventive Measures against Cancer: Current Trends and Directions

**DOI:** 10.3390/foods11213349

**Published:** 2022-10-25

**Authors:** Anuva Talukder Trisha, Mynul Hasan Shakil, Suvro Talukdar, Kobun Rovina, Nurul Huda, Wahidu Zzaman

**Affiliations:** 1 Department of Food Engineering and Tea Technology, Shahjalal University of Science and Technology, Sylhet 3114, Bangladesh; 2 Faculty of Food Science and Nutrition, Universiti Malaysia Sabah, Kota Kinabalu 88400, Sabah, Malaysia

**Keywords:** catechins, anti-cancer agent, health benefits, cancer prevention, bioactive compounds

## Abstract

Cancer is exerting an immense strain on the population and health systems all over the world. Green tea because of its higher simple catechin content (up to 30% on dry weight basis) is greatly popular as an anti-cancer agent which is found to reduce the risks of cancer as well as a range of other diseases. In addition, several in vitro and in vivo studies have shown that green tea possesses copious health benefits like anti-diabetic, anti-obese, anti-inflammatory, neuro-protective, cardio-protective, etc. This review highlights the anti-carcinogenic effects of green tea catechins integrating the recent information to gain a clear concept. Special emphasis was given to the effectiveness of green tea polyphenols (GTP) in the prevention of cancer. Overall, green tea has been found to be effective to reduce the risks of breast cancer, ovarian cancer, liver cancer, colorectal cancer, skin cancer, prostate cancer, oral cancer, etc. However, sufficient information was not found to support that green tea consumption reduces the risk of lung cancer, esophageal cancer, or stomach cancer. The exciting data integrated into this article will increase interest in future researchers to garner more fruitful information on the relevant topics.

## 1. Introduction

Tea, derived from *Camellia sinensis*, is now one of the most consumed beverages in the world. Based on the level of fermentation, tea is generally of three types: green tea (unfermented), black tea (fermented), or oolong tea (partially fermented) [1]. A study performed in 2010 shows that the black tea consumption rate in the current world is very high (almost about 78%, especially in the USA, Europe, and Western Asia). Green tea is getting popular among the consumers too (comprises 20% of the world consumers especially in Japan and parts of China). The remaining 2% like to consume oolong tea (mostly drunk in Southeastern China and Taiwan). In the recent years, tea has gained its increased popularity because of its delicate taste, impressive aroma, stimulating impacts and many health benefits [2]. 

More than 30 countries are now involved in tea production successfully. Observing the huge popularity of tea recently, researchers got interested in thinking about the health potentials of this popular beverage [3].

It is presumable after several studies that tea has played a significant role as a traditional herbal medicine [4,5]. Although green tea is consumed less than black tea, it possesses much greater polyphenolic contents [3]. 20–30% of dry weight of green tea is tea polyphenols [6]. Fermentation in tea is defined as the oxidation of catechins in the presence of polyphenol oxidase enzymes with the end products being theaflavin, thearubigins, theasinensins, and theabrownins [7]. Although the fermentation products in black tea demonstrate several biological activities, the antioxidant activity is higher in green tea than that of black tea [8]. Green tea contains several beneficial compounds. Table 1 summarizes the major beneficial constituents found in tea. Catechin is one of the most important compounds found in tea and demonstrates strong antioxidant activity by neutralizing free radicals.

**Figure 1:**
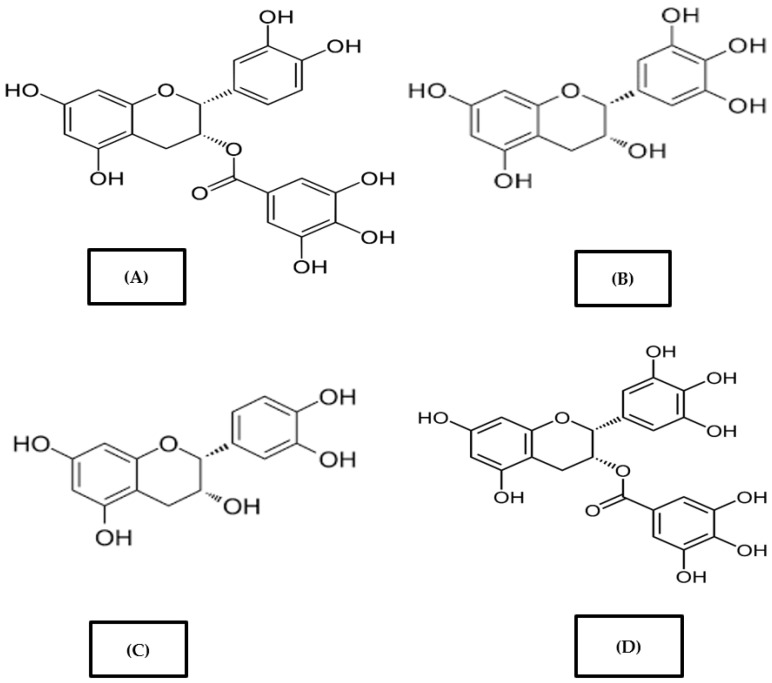
Chemical structures of major polyphenols present in green tea: (**A**) Epicatechin gallate, (**B**) Epigallocatechin, (**C**) Epicatechin, (**D**) Epigallocatechin gallate.

Four types of principal catechins are found in green tea whose structures are demonstrated in Figure 1 [11]. Epigallocatechin gallate (EGCG) comprises 59% of all catechins, Epigallo catechin (EGC) makes up 19%, Epicatechin-3-gallate (ECG) comprises 13.6%, and finally Epicatechin (EC) comprises 6.4% catechins [12]. It is assumed that among the catechins, gallates (ECG) and gallocatechins (EGC and EGCG) possess more healthy benefits [13]. In addition, green tea has several other components including polysaccharides, alkaloids (Caffeine), free amino acids, polyphenols, etc. These components are mainly responsible for different functionalities of green tea [14]. Other than the bioactive compounds, green tea also contains caffeine and theanine that attribute significantly to the sensory qualities and bring attention of the consumers [15].

The significance of EGCG against cancer has been studied for more than 30 years [16]. For example, it is thought that EGCG can boost antitumor immunity and tighten the bonds of the cell membranes as well as inhibit the signaling of various cancer receptors, thus showing beneficial health effects. The polyphenolic structures with available H and O atoms make green tea able to donate weak hydrogen bonding so that these catechins may bind to cell proteins and nucleic acids to contribute to prevent cancerous effects. The reduction-oxidation reaction (Redox) balance is very crucial for human health. The reactive oxygen species (ROS) are the products of normal cellular metabolism that are proved to be responsible for showing cancerous effects in the human body [17]. Tea polyphenolic contents may act as chelators of metallic ions and can prevent the reactive oxygen species (ROS) to form in our body. Again, EGCG can react with the ROS to extinguish free radicals from our body [18]. Several epidemiological studies have reported that green tea provides a significant protection against cancer occurred at different organ sites in human beings [3]. All these activities of GTPs make them effective to act as cancer preventive agents. The superior mechanisms for which green tea is considered as a better natural preventive agent are like inhibition of cell apoptosis, cell-cycle arrest, and suppression of cell proliferation, etc. [19].

**Figure 2:**
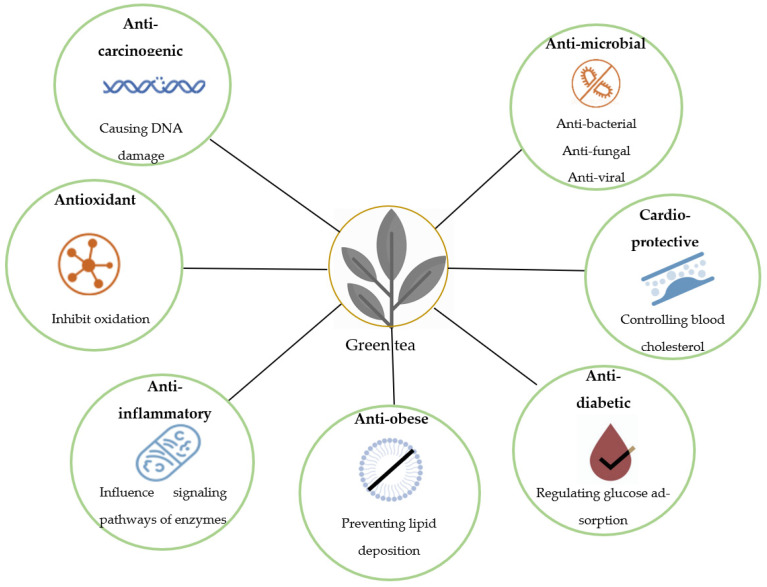
Prime health benefits of green tea.

Initially, green tea is prepared by steaming (Japan) or roasting (China) the dry tea leaves and thus, polyphenol oxidase enzyme activity present in tea leaves is inhibited resulting in the end of polyphenol oxidation during further processing [4]. Thus, the quality of polyphenols is retained which makes green tea much healthier than black tea in which polyphenol oxidase enzyme converts the polyphenols to other compounds (theaflavin and thearubigin, responsible for black tea color and flavor) resulting in a reduction of polyphenols [3]. Green tea demonstrates several health benefits that are summarized in Figure 2 [20,21,22,23].

Green tea are enriched with bioactive compounds, especially polyphenolic contents with their antioxidant properties. A cup of green tea contains approximately 300 mg of catechins, i.e., a cup of green tea brewed with 2.5 g of dry tea leaves poured in 0.25 L of water carries almost (30–40%) of catechins whereas the amount of catechins is (3–10%) in black tea [4]. Green tea infusion carries almost 1 g/L catechins that serve several health benefits while the amount of catechins becomes half in case of black tea [9]. A number of studies declared that green tea has anti-cancer activity, efficacy against cardiovascular diseases and diabetic or neurological problems [24]. For example, one cup of green tea has 200 mg of EGCG having fruitful medicinal effects on several kinds of cancers. People drinking 3–5 cups of green tea per day intake 250 mg of catechin which strengthens their health [24].

Cancer is creating a malignant effect on human health and is becoming the second leading cause of human deaths after cardiovascular diseases. Cancer develops preliminary symptoms in human body along with a latish diagnosis and restricted treatment policies resulting in facing problems to control the mortality rate [25]. A recent survey performed by Global Cancer Statistics in 2020 revealed an alarming result that almost 19.3 million new cancer cases and 10 million cancer deaths occurred in the year of 2020. Statistically, the highest death rate is found in the case of breast cancer (approximately 11.7%), followed by lung cancer (11.4%), colorectal cancer (10%), prostate cancer (7.3%), stomach cancer (5.6%) and so on [26]. Several studies have reported that green tea can work very well against cancer. Although some of the literature has reported inconclusive results about the effectiveness of green tea against stomach cancer—and that a high dose of hot green tea might cause stomach cancer [27]—numerous articles reported the anti-cancer activity of green tea. This review will discuss the recent findings on the activity of green tea polyphenols (GTP) against cancer and provide directions for further studies.

## 2. Search Approach and Study Choosing

### 2.1. Search Approach

**Figure 3:**
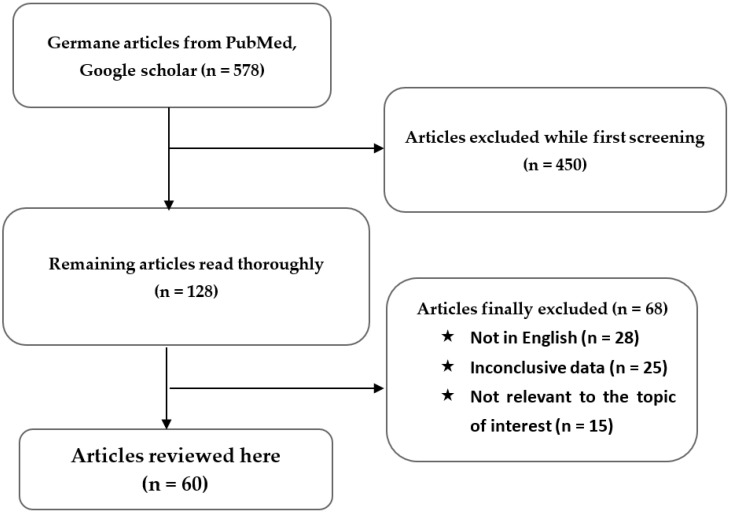
Flow diagram of the literature search and study choosing procedure.

To initiate collecting review articles related to the selected title, widely used search tools like Pub, Med, and Google Scholar were used for literature search. After searching for the first time, 578 results were found on the relevant topic performed from the origin to the present. After screening, few articles were selected that were to be summarized from 2000 to 2021 depending on the necessity level to perform this review. This paper reviewed almost 60 articles published currently and the literature search approach is summarized in Figure 3.

All the selected articles were read thoroughly from the beginning to the end to understand. To find the necessary articles, the keywords used were “tea polyphenols” or“ green tea” and “cancer” or“ green tea polyphenols” and “cancer prevention” or “catechins related to cancer” or “EGCG to cancer risk” or effect of “*Camellia sinensis* on cancer”.

### 2.2. Inclusive Criteria

The studies related to the discussion about green tea polyphenols and the prevention of cancer risk or treatments of cancer were included in this review. Brief descriptions about the effects of tea on several cancer types were also added. Only previously performed observational studies were summarized in it. Already proven information in recent times were discussed instead of using any prognosis data in this article.

### 2.3. Exclusion Criteria

Articles written in regional languages were fully avoided. Literatures containing insufficient data on the relevant topic or failing to describe the positive, healthy effects of green tea were totally excluded. Articles overviewing case-control studies, population-based data of animal models, etc., were not included in this review. Studies of cancer forerunning agents were skipped while performing this review. Meta-analysis related studies were not considered in this case descriptively. Papers in which inconclusive results were found were not used to review.

## 3. Mechanistic Studies on the Activities of EGCG against Cancer Cell Lines

**Figure 4:**
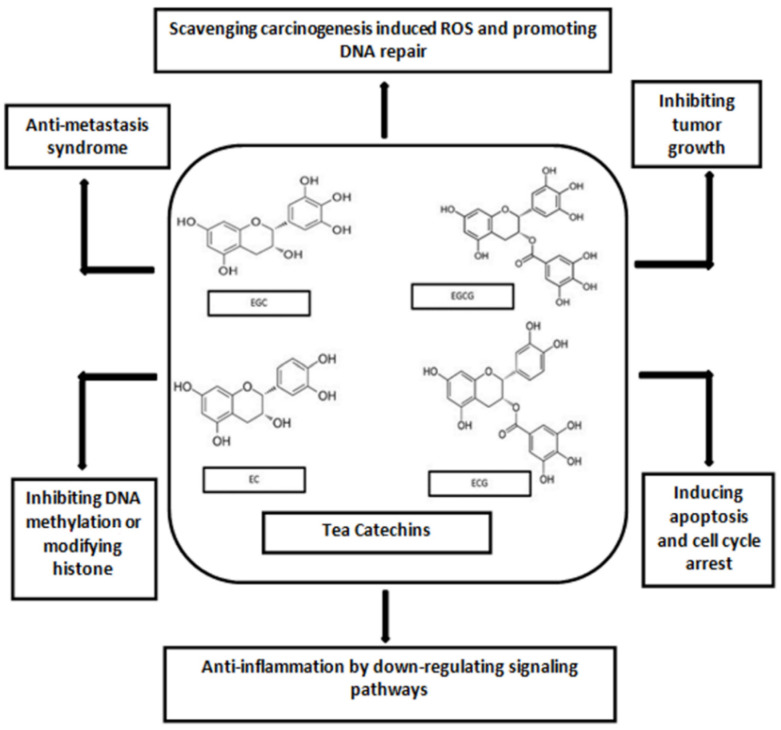
Mechanism of green tea catechins against cancer.

The principal tea polyphenol, EGCG has been examined more due to its better cancer preventive mechanisms. Tea polyphenols are shown to have their effects on increasing the levels of enzymes detoxifying carcinogens in human cells [28]. The various components of tea are seen to impede carcinogen-induced DNA damage. When green tea is drunk, ROS may be produced by EGCG after entering the cells and this event may cause oxidative damage in cancerous cells. EGCG are found, in several studies, to bind to different target proteins in the cells causing cell apoptosis [29]. Figure 4 demonstrates the mechanisms of green tea polyphenols in cancer prevention [22,30].

## 4. Studies on Green Tea and Human Cancer

Until now, the association between green tea and cancer prevention has been reviewed by numerous researchers. It is now believed that green tea extracts, especially pure EGCG, are greatly capable of protecting human beings from carcinogenic effects through different mechanisms. Therefore, this review was performed to summarize the roles of green tea polyphenols on reducing the risk of cancer without causing any side effects. The results found after overviewing the relationship between green tea polyphenols and several types of cancers are discussed below:

### 4.1. Breast Cancer

Breast cancer [20] is a heterogenous disease seen in women though it can affect both males and females [24,31,32]. It is deemed to be the most commonly diagnosed type of cancer constituting approximately 25% of all diagnosed cancer types [33]. There has still been a steady decrease in breast cancer occurrence and mortality cases for the last few decades. Major factors influencing the risk of breast cancer: improper diet, inadequate daily nutrient intake, obesity and inactive life pattern, smoking and drinking excessive alcohol, high breast density, previous family background of breast cancer, etc. [33].

Three successful treatment processes such as chemotherapy, radiation therapy, and hormonal therapy are followed at present against breast cancer [32]. However, because of serious side effects for multidrug resistance and tumor recurrence, scientists are now trying to apply some natural medicines to prevent or treat breast cancer patients. EGCG has a positive impact on tumor glucose metabolism system. Breast cancer 4T1 cells are treated with the help of EGCG which inhibits abnormal cell growth and thus, apoptosis (a process of cell death to help the body get rid of its unwanted cells) is induced by activation of caspases 3,8,9 modulation of apoptotic associated genes and so the mitochondrial depolarization process is promoted. EGCG inactivates a Beta-catenin signaling pathway to stop the cancerous cell growth [31]. In addition, GTCs have been shown to deduct cell proliferation through the transition of cell cycle advancement [34].

A study performed in 2014 also found a cooperative role of EGCG in treating breast cancer cases [27]. These findings about the relationship between green tea and breast cancer prevention are displayed in Table 2. A study performed in the last decade in Singapore on postmenopausal women reported that regular green tea drinkers had less mammographic density (19.5%) than non-tea drinkers (21.7%) [35]. The high antioxidant activity of green tea might boost women’s immunity and help them burn extra fat and calories thus resulting in a reduced breast size. Green tea has also been found to reduce the breast tumor size. Thus, green tea is now popular because of its efficacy against breast cancer as well as treatment due to its greater potential. Another in vitro study reported that a polyphenolic complex of catechins and lysine is found to affect positively in breast cancer cell lines. This nutrient complex exerts selective anti-migratory effects through interfering with glucose uptake by tumor cells.

The relationship between green tea polyphenols and cancer risk was also experimented in a dose-regarded manner and menopausal status also. A study showed that stage 1 and stage 2 breast cancer women who drank more than 5 cups of green tea per showed lesser risk compared to those who took four or less cups of tea daily [32]. According to a study performed among 472 women, it was found that premenopausal women who drank at least 5 cups of green tea showed less recurrence of breast cancer in the case of stage 1 and 2 cancers (16.7%) than those who consumed less than 4 cups per day (24.3%). It has also been reported that in case of stage 3 breast cancer, patients who drank regular green tea didn’t show any reduction in cancer risk owing to more genetic changes in the cells than stage 1 or 2 [36]. Women who were under the age of 50 who drank 3 or more cups of tea were 37% less likely to be affected with breast cancer than non-drinkers of green tea. Regular intake of green tea, even at a lower amount over the years in the case of postmenopausal women, showed a reduced risk of breast cancer. A study conducted in 2007 proclaimed that women consuming ≥ 250 g/year green tea extract may have a lower risk of getting breast cancer [37].

**Table 2:** Effects of green tea polyphenols in preventing cancer.

Target Organ	Mechanism of Action	Study Type	Reference
Breast	Inhibition of cell growth and enzymatic activities, Development of mitochondrial depolarization	In vivo	[38]
Lung	Decrease in the mRNA and protein levels, Inhibition of tumor growth	In vitro, in vivo	[28,39]
Colon-rectum	Induction of nuclear translocation, reduction in tumor multiplicity and size	In vitro	[40]
prostate	Provision of resistance against oxidative stress, Induction of kinase enzyme to inhibit cancerous cell growth	In vitro, in vivo	[41,42]
Stomach	Stimulation of protein kinase, interference with cell signaling pathways	In vitro, in vivo	[43]
liver	Reduction in cell inflammation	In vivo	[44]
Skin	Invasion of melanoma cells, inhibition of melanoma tumor growth	In vitro	[45,46]

A successful study has been performed to prevent breast cancer with a nutrient mixture comprising of lysine, proline, ascorbic acid (Vit C), and green tea leaf extract. The positive result of that study inspired the scientists to use the mixture to treat cancer and the research is still on progress [24].

### 4.2. Lung Cancer

Nowadays, lung cancer accounts for the second highest death globally [23]. The risk factors responsible for this type of disease are (a) tobacco smoking, (b) taking regular dietary supplements, and (c) previous radiation therapy of lung, etc. Among them, chain tobacco smoking is the compelling reason for most of the cases and due to lack of multidrug resistance, fewer number of patients get diagnosed from lung cancer.

From the previous studies, it has already been proven that green tea polyphenols contain significant potential against cancer. Therefore, numerous studies were performed to understand how effective green tea polyphenols are against lung cancer. Forty-five articles from the literature were found on the relevant topic while searching primarily. Among those, 6 studies were selected to gather proper information on it.

Recent studies have proven that green tea catechins have tumor growth inhibitory impact and anti-proliferative effects against lung cancer making green tea health-effective in the case of those patients [9]. A 2020 in vitro study has already reported that EGCG carries significant potential with existing therapies in cancer patients by blocking protein kinases [31] without creating any side effect among them [32]. An experiment conducted on Chinese women showed that a 35% reduction was found in the case of those women who drank green tea on a regular basis with respect to non-drinkers of green tea [47]. Tea catechins protect the body cells from DNA damage as measured by urinary 8-hydroxydeoxyguanosine among native smokers. EGCG protects the patients by inducing apoptosis in LC cells in their bodies [9].

Another study reported that EGCG can inhibit tumor growth receptors and stop nicotine-induced element migration in cell disrupting LC development [31]. These mechanisms of green tea polyphenol and EGCG in preventing lung cancer are demonstrated in Table 2. Several studies reported that green tea polyphenols have chemo preventive effects even among regular cigarette smokers by blocking the cigarette-induced elements [9]. If non-drinkers start consuming green tea regularly, they can be protective against lung cancer. If the consumption is increased into 2 cups per day by them, it could possess an 18% lesser risk of developing lung cancer [48]. It has already been stated that cigarette smokers receive a 31% decrease in LC development after drinking decaffeinated green tea at the rate of 4 cups per day for 4 months whereas no mentionable change was visible in case of smokers taking black tea evidencing strongly that antioxidation of green tea has a little role in reduction of risk of lung cancer [32]. A dose-response study to develop a relationship between green tea intake and prevention of lung cancer was conducted on women in Shanghai, China. The study revealed that a 35% reduction was counted to lessen the lung cancer risk in those who drank green tea regularly than those who never drank tea [49].

Though maximum studies have said that green tea is useful in preventing the risk of lung cancer and its catechins can act as powerful agents, several studies are postulating that green tea along with black tea may often increase the risk of lung cancer. For example, in case of treating lung cancer patients who take Bortezomib regularly, it is found that green tea may have some negative impacts on them as EGCG remaining in green tea may bind with the supplement and block its therapeutic effects and thus make it ineffective [32].

### 4.3. Colorectal Cancer (CRC)

The third leading cancer type in case of mortality rate is becoming one of the major concerns of the specialists [33]. This kind of cancer starts spreading from colon or rectum, the final parts of our digestive tract and becomes dangerous day by day because it is comparatively hard to detect and so, the number of deaths is increasing gradually. Colorectal cancer actually begins with the creation of adenomas, along with varying levels of malignant potentials, finally leading to adenocarcinoma [50]. Several epidemiological studies postulated adverse lifestyle factors of human beings for the risk of this carcinogenesis. The factors considered to be responsible include excessively high BMI content (Obesity), alcohol consumption, less physical work, and lower metabolism capacity, etc. [51].

Inspired by the positivity of green tea against colorectal cancer, various studies were conducted to evaluate the relationship between GTPs and the risk of colorectal cancer. 445 results were found while searching for the first time on this topic. From those, five articles were selected for this study. All the research conducted described EGCG as the best effective agent against CRC among tea catechins. Basically, EGCG can influence the growth reduction of tumor cells of small intestine in a dose-dependent way helping the human body prevent cancerous effects [29]. However, one study performed in Japan could not find a significant association between green tea and the risk of colorectal cancer [52]. A recent study has reported that EGCG has a role in radiation also. According to that study, EGCG with the help of radiation technology may inhibit cell proliferation and thus help the tumor cells be inactive [24,40]. When the colorectal cancerous cells are treated with green tea extracts, then EGCG help inhibit cell proliferation, induce apoptosis of cells and so on [53]. The protective effects of EGCG against this colorectal type of cancer are discussed in Table 2. Among the studies performed, most of the studies showed inconclusive results. Some studies revealed that women drinking green tea regularly responded better than men against CRC. A cohort study which was performed among Chinese women drinking more than 5 cups/day noted that those women possessed lesser risk in comparison with non-drinkers [54].

### 4.4. Prostate Cancer (PC)

In the case of the men of Europe, North America, Australia, African countries, prostate cancer is very common nowadays [31]. Globally, the number of patients affected by PC and death rates are increasing day by day. Factors influencing the possibility of prostate cancer are age, high BMI content and lower physical activity, habit of regular cigarette smoking, alcohol intake, etc. [33,55]. A recent study has shown that the risk of this cancer is seen to be increased in Asian men who have started eating more red meat and fatty foods instead of eating soy, fish, fruits and vegetables, tea, etc. [33].

It is seen that patients can recover early from prostate cancer by regular screening but to control the upcoming dreadful situation of it, scientists are thinking about prevention or treatment of prostate cancer with some natural agents to ignore the side effects of artificial supplements [31]. GTPs may play a vital role in this case. From different studies, it is evident that GTPs show anti-cancer impacts as these have the ability to induce DNA methylation. Several studies reported that EGCG, one of the key components of green tea, can resist cell apoptosis as the molecules related to apoptosis are influenced by EGCG [56].

Another recent study has declared that the production of prostate cancer creating cells is decreased by the activity of matrix-metallo proteinases (MMP) and their tissue inhibitor (TIMP-3). EGCG intercedes the reactivation of TIMP-3 phases which finally acts by inhibiting prostate cancer development [31]. The inhibiting properties of green tea polyphenols against prostate cancer are described in Table 2. Moreover, a dose-dependent study was also performed to observe the efficacy of green tea against the development of prostate cancer in men. The study declared a better situation in the case of those men who consumed almost ≥ 5 cups green tea/day in comparison to those who drank green tea less than 1 cup/day [47].

Green tea flavonoids may also induce apoptosis in PC cells [56]. Thus, GTPs help in cancer treatment by arresting carcinogenic cell cycle production and resisting cell apoptosis which stops cancer cell making [31]. Human clinical trials are still going on to declare more proper data on the topic.

### 4.5. Stomach/Gastric Cancer

Due to an irregular daily diet, in today’s world the number of cases of gastric cancer is increasing day by day. Considerable factors for the final diagnosis of gastric cancer are lifestyle habits (very hot food eating habit), family history of stomach cancer and peptic ulcer, alcohol intake and smoking, intake of coffee and black tea excessively, less physical exercise, and a weak metabolism system, etc. [44].

To summarize the relation of green tea polyphenols to gastric cancer prevention and treatment, initially 268 results were found while searching and from those, three recent articles were selected. According to the studies, green tea provided better protection to females than males, but significant protective effect was not found in the case of non-smoker males or females. Data found on the association between green tea catechins and reduction in the risk of gastric cancer development are still inadequate. The effects of green tea catechins or other metabolites against stomach cancer were tested by determining their concentration in urine [47]. A meta-analysis performed recently declared that EGCG can block multistage carcinogenesis by stimulating protein kinases [43]. It was found that ECG can also interfere with multiple cells’ signaling pathways and it has several cellular targets which make it perfect to reduce the risk of gastric cancer.

The influences of GTPs on the corresponding subject were also evaluated in a dose-response manner. By considering several studies, it was understood that persons intaking lower doses of tea cannot be totally protected from the risk of this cancer [57]. For example, a study compared the women in China drinking <1 cup/day found an almost 20% decrease in cancer risk [47]. On the contrary, women with a greater dose, almost 5 cups or more per day, showed a 26% reduction in risk of stomach cancer [57]. In addition, six studies were conducted in Japan with a cup of green tea infusion of 100–120 mL and unfortunately, four of these showed no relation to green tea with the prevention of gastric cancer taking that infusion regularly [58].

Another cohort study found the evidence that when green tea was drunk at the rate of (≤4 cups/day), it was unable to bring about any positive sign against gastric cancer. At the same time, the study revealed that they found evidence that green tea showed a gradual increase in preventing cancerous effects when more than 5 cups/d were drank [57]. Finally, it was declared that persons drinking green tea almost ≥35 g/month showed better prevention against stomach cancer at a mentionable amount. A mentionable decrease in stomach cancer risk was statistically significant only when green tea was consumed at the rate of ≥7 cups/day [50]. The best result was found in those females who took generally 10 or more cups of green tea within a day by performing a hospital population control test [57].

### 4.6. Liver Cancer

Recent studies have reported that people in the Asian region are in high risk of liver cancer familiar with the name “Hepatocellular Carcinoma (HCC). The factors which stimulate our body to be affected by liver cancer include alcohol and high caloric food consumption, cigarette smoking, obesity, long-term diabetes, Aflatoxin contaminated food, etc. [44]. The most serious risk factors responsible for this cancer are Hepatitis B virus infection or Hepatitis C virus infection.

According to doctors, regular monitoring over the patients, vaccination against Hepatitis viruses, and proper antiviral treatment can protect our people from liver cancer [44]. But it is always better to look for natural remedies to protect ourselves. That is why the connection between green tea polyphenols and liver cancer risk prevention was eagerly studied by the researchers. To summarize the findings of the previous studies, only eight results were found on this selected topic while searching for literature.

Studies declared that green tea is more effective in women than men. Women who consumed green tea showed a lower mortality risk of liver cancer compared to women who did not consume green tea [3]. Most of the studies did not find any relation between green tea and the prevention of liver cancer risk in men [47]. Green tea proves itself more effective in preventing liver cancer when it is consumed at a higher rate. EGCG found in green tea may induce hepatic toxicity at high regular intakes. Women drinking 5 or more cups of tea daily were found risk free from liver cancer by 22%. On the contrary, no connection was found with lower intake of green tea by women who drank 1 cup/day [44]. While considering the previous study results, it was found in most cases that regular green tea consumption may act more well on fatty liver disease and other liver disorder cases than liver cancer [59,60]. The results of these research indicates that green tea has beneficial influences in preventing oxygen free radicals created due to hepatocyte lethality, suppressing hypo-polysaccharide induced liver injuries, inhibiting carcinogenic toxin induced DNA damage in the liver and so on, which is shown in the picture below. Another study showed that oral supplementation of green tea polyphenols at the rate of (500–1000 mg/day) can promote the detoxification of aflatoxin, thus resisting oxidative DNA damage and playing a vital role in preventing liver cancer [47].

### 4.7. Skin Cancer

In today’s climatically changing world, skin cancer is the most familiar type worldwide. Skin cancer is of two classes including non-melanoma skin cancer (NMSC) and Melanoma. Non-melanoma cases are further classified into two forms like Basal cell carcinoma (BCC) and Squamous cell carcinoma (SCC) [9,12]. Both of the classes are seen among the people of New Zealand and Australia [61]. In spite of having numerous risk factors behind developing skin cancer, the major risk factor is absorption of UV ray through absorbing direct sunlight [31,61]. The level of global warming is increasing day by day as well as the number of skin cancer patients, especially melanoma cases. But therapeutic treatments for melanoma cases may have severe side effects [9,12]. Therefore, scientists worldwide are thinking about handling this situation by applying natural remedies [9]. To give a short summary on the relevant topic, almost 120 results were found while searching. Then 5–6 studies were selected to overview the topic.

A 2019 study reported that people who intake tea caffeine content more are affected by skin cancer by 43% less than non-consumers of tea [24]. Better anti-carcinogenic power is seen against skin cancer by green tea polyphenols and flavonoids [9]. A recent study has declared that human skin can be protected from UV damage by applying green tea extracts on skin [61]. From epidemiological studies it has been reported that direct sunlight exposure causes direct DNA damage in skin by forming cyclobutene pyrimidine dimers (CPDs). GTPs play a vital role in inhibiting the above formation of CPDs by the use of sunscreen applied with green tea extracts [62]. The number of sunburn cells is seen to be reduced in those skins treated with GTPs and thus skin cancer related difficulties are also reduced [61]. EGCG along with its anti-inflammatory and anti-cancer properties can prevent the growth of skin tumors [9]. Treatment of skin cancer with EGCG influences mRNA expression of previously silenced tumor suppressor genes thus lessening the tumor cell production incidence. EGCG and GTPs may show effective chemo preventive properties against skin cancer [61]. In this case, tea polyphenols play their major role by repairing DNA to return the cells back to their healthy state. These present understanding about the mechanism of green tea polyphenols in preventing skin cancer is shown in Table 2.

### 4.8. Pancreatic Cancer

Due to irregular and improper dietary habits of individuals, the number of pancreatic cancer cases is increasing day by day. It is seen in previous research that over 60% of pancreatic cancer cases are found in the developed countries of the world [63]. The factors having a role behind creating this type of cancer are tobacco smoking, diabetes, obesity and physical inactivity, previous difficulties of the pancreas (inflammation), family background of this type of cancer, age, and so on. As there remains little possibility of finding any prediction about the occurrence of pancreatic cancer, this disease is diagnosed at the advanced stage that makes it as one of the most severe types [12]. To reduce the death rate, a strong preventive method is crucially needed. Green tea polyphenols may help us in this case.

Several studies are conducted by the researchers to evaluate the efficacy of GTPs in preventing as well as treating pancreatic cancer. To summarize findings from previous studies, eight articles were found after the literature search. Generally, it is proven that green tea can play a vital role in women compared to men. For example, several studies showed that the number of women drinking green tea in large amounts and thus showing better protection against cancer is almost half compared to the women consuming a lesser amount. The opposite is found in the case of men. The number of men with a longer duration of tea drinking and a lesser risk of cancer is only 37% compared to the non-drinkers. A study reported that people drinking larger amounts of green tea regularly (i.e., 7 or more than 7 cups of green tea per day) showed better results than those who drank less than 1 cup of tea [64]. Due to having the power of suppressing the migration and invasion power of cancerous cells into the pancreas, EGCG is considered as a better natural agent for preventing cancer by numerous articles. Another article declared that EGCG can alter metabolism properties of cancer cells by inhibiting the expression of an enzyme associated strongly with this cancer [12].

Again, a case-control study performed among Japanese people showed no association between GTPs and the prevention of cancer risk, whereas a population based study performed in China found an inverse association between them [3]. A study performed in Japan could not unfortunately show any association between green tea consumption and reduction in the risk of pancreatic cancer [65]. Positive and negative impacts on health were revealed by the researchers while detecting the effectiveness of GTPs in the prevention and treatment of pancreatic cancer.

### 4.9. Oral Cancer or Dental Problems

In the present world, oral cancer is presenting a new challenge to scientists. The risk factors for developing oral cancer are regular alcohol drinking and smoking, excessive sunlight exposure, etc. The treatment cost of this cancer is relatively high, making it generally unaffordable for many people [3,47]. On the contrary, due to rapid climate change, the rate of new cases is upgrading significantly. That is why it is better to think about preventive agents to protect human beings from oral cancer or dental problems. Green tea constituents may act effectively in this case.

Very limited studies have been performed to identify the relationship between green tea and oral cancer prevention and treatment. This corresponding article aims to summarize the findings of those relevant studies. A case-control study revealed that people who drank 1 or greater than 1 cup of green tea on a day possessed 37% reduction in the risk of oral cancer in comparison to non-tea drinkers [3]. However, better results were found in the case of those who drank at least 5 or more than 5 cups of tea per day than those who drank less than 1 cup of tea daily [47].

According to a 2013 study, it was found that patients regularly taking green tea extracts in the form of supplements were seen to suppress the harmful effects of malignant tumors. For instance, a study performed with almost 59 patients given 3 g of a mixed green tea extract per day presented the information that among the patients, approximately 37.9% showed reduced wound size whereas only 3.4% showed increased tumor size unfortunately presenting an inverse relationship between GTPs and oral cancer risk [47]. While studying about the impact of principal constituents found in green tea and EGCG against oral cancer, it was found that EGCG protects dental materials from bacterial infection thus keeping them well and safe. It is evident in a study that EGCG can act as an obstruction to prevent dental cell proliferation and migration of epithelial cells as well as play a major role in regenerating healthy oral and dental cell tissues. Epicatechins (EC) enhance surface strength thus making dental pulp and bones strong [66].

However, a recent study has reported that regular usable toothpaste containing EGCG may protect dental cells from inflammation thus giving the patients more comfort and reducing cancer risk due to its antimicrobial properties [66].

### 4.10. Ovarian Cancer

At present, it causes the highest death in women. The factors influencing the risk of ovarian cancer include age, previous family history, being excessively obese, becoming pregnant at an older age, etc. Due to a limited or no possibility of prognosis before the advanced stage, the mortality rate of this type is increasing enormously [67]. That is why searching for a better preventive agent has become one of the challenges for scientists. It is estimated that green tea polyphenols may help researchers prevent the risk of ovarian cancer in women.

Much research has been performed until now to finalize the effects of green tea in the prevention and treatment of ovarian cancer. To overview those studies, eight matched results with the corresponding topic were found in literature search.

A study performed in China reported that women drinking at least 1 cup of tea daily lived longer than non-drinkers. According to another study, a 40% reduction was estimated in risk of ovarian cancer in women consuming 1 or greater than 1 cup of tea on a regular basis [68]. Though having some controversial data, several studies claimed that caffeinated green tea showed better effect against ovarian cancer compared to the decaffeinated type [67]. It has also been reported that EGCG can stop DNA damage and tumor development in ovarian cells due to its antioxidant characteristics.

Several studies found that EGCG can play a significant role in demobilizing enzymes named Urokinase and Telomerase responsible for cancerous effects in women’s body and thus may help to defeat cancer. It has also been stated that EGCG along with theaflavin in black tea may hasten the process of apoptosis and cell-cycle arrest [67]. An in vitro result reported that an infusion of green tea at the rate of mixing (2–4) g tea leaves with 350 mL appeared to have better inhibitory effects on ovarian cancer cell lines in comparison to the infusion of 250 mL. Unfortunately, greater green tea intake may possess a negative impact on the occurrence of cancer, i.e., drinking 4 or more than 4 cups per day [69]. In one study, it was found that increasing the green tea consumption rate after post-diagnosis may cause fast survival from ovarian cancer [70]. In western regions such as Australia, it was found that the rate of green tea consumption belongs to 1 cup of tea per week. With this lower proportion of intake, detection of the efficacy of green tea in cancer prevention was quite impossible and thus, most of the studies were performed in Asian countries such as China and Japan [68].

### 4.11. Bladder Cancer

Due to unhygienic diet, the number of bladder cases is also increasing at a threatening rate at present. The factors considered for developing bladder cancer include excessive smoking, harmful chemical exposure from the workplace, drinking water contaminated with arsenic, age, family history, etc. As a poor level of prognosis is identified in initially detecting this cancer, prevention is better to be protected from it [71]. The literature search for describing the effectiveness of GTPs against bladder cancer included almost seven matched articles.

Clear evidence has been found from research that due to huge tea consumption, the number of cases of bladder cancer is relatively lower in Asian regions than in the United States and Europe [72]. According to several studies, both males and females are at a significant risk of bladder cancer [71]. Urinary bladder cancerous cells which are treated with EGCG can be easily detected with expressed genes and miRNA interactions [73]. Another study found that higher concentration of ECG and EGCG intake (60 microgram per mL) can inhibit bladder cancer cell proliferation [71]. According to another study, it was found that greater exposure to carcinogens in urine may cause cancerous effects. Then tea intake at a higher amount may dilute this urine and increase the frequency of urination thus reducing bladder cancer risk [74]. It was found in another study that ODC (Ornithine decarboxylase)—an enzyme playing an important role in tumor development in human bladder cells by DNA, RNA, and protein synthesis—lost its activation power by green tea polyphenols and became unable to create carcinogenic effects on human bodies. Green tea extracts also play a vital role in preventing bladder cancer by interfering with carcinogenic acting components inside human bladder cells [72,75].

A clinical study performed by the researchers reported that men who had already been affected by bladder cancer could gain an almost 5-year survival rate compared to those who never drank green tea. Again, many researchers thought that black tea along with green tea in a powder form can work better against bladder cancer [71]. It has also been suggested that a mixture of black tea, decaffeinated tea, green tea along with other type of herbal teas may have more effective roles in bladder cancer risk reduction rather than green tea alone [76].

Though most of the studies found no strong association between tea drinking and reduction in the risk of bladder cancer, a study which was conducted in the Canadian region was among the studies which believed that tea has the power to reduce cancerous effects. Consuming greater than or equal to five cups of black or green tea per day can produce a 30% reduced risk of urinary bladder cancer with non-drinkers [3].

### 4.12. Esophageal Cancer

This type of cancer occurs more in men compared to women. According to population-based studies, green tea drinking at a moderate level can protect women better from this form of cancer. The risk factors of esophageal cancer are age, smoking and alcohol drinking, obesity and physical inactivity, improper diet, consumption of salted food regularly, etc. This type of cancer may be of two kinds such as squamous cell carcinoma and adenocarcinoma [48].

According to most of the previous studies which were performed in the nineteenth century, the stronger the infusion of tea and the hotter the tea, the greater the risk of esophageal cancer. Green tea intake at a very high temperature might cause serious damage to the esophageal cells thus possessing a greater risk of developing esophageal cancer [3]. It has also been reported that hot tea could affect esophageal mucosa directly or indirectly which could be responsible for chronic thermal injury in the esophagus resulting in enhancing the risk from exposure to intraluminal carcinogens like polycyclic aromatic hydrocarbons [77]. However, the recent studies reported a significant association between green tea intake and reduced cancer risk.

A population-based study performed in Jiangsu Province, China notified that regular green tea consumption could possess a lower risk in esophageal cancer in comparison with no consumption [78]. Chinese men are greatly affected by this disease [79]. A Chinese population-based study has revealed that green tea showed its better performance in reducing the risk of cancer in those men who were never involved with cigarette and alcohol consumption [47]. Recent studies have shown that tea consumed at least at a temperature of less than or equal to 65 °C may create reduced risk [80]. On the contrary, many studies reported that although green tea can decrease the risk of cancer development, black tea can enhance the risk [81]. According to another study, women consuming ≥150 g/month green tea leaves are associated with the reduction in cancer risk [37]. A study based on tea infusion type consumed along with maintaining proper temperature showed us that the infusion of green or black tea made with less than 300 g/month may significantly lessen the risk [81].

It was also found in a study that green tea extracts rich in EGCG can inhibit cancerous cell cycle production which promotes its degradation. EGCG may also resist cell proliferation and therefore helps in reducing the cancerous effects in esophagus cells [74]. Table 3 summarizes the epidemiological studies of the relationship between green tea consumption and human cancer risk.

## 5. Discussion and Limitations

After this review, it can strongly be declared that green tea contains several compounds that have significant anti-cancer activity. EGCG, especially, is very efficacious to prevent cancer [85].

In this study, it was seen that regular green tea intake had better treating power against breast cancer in the case of premenopausal women whereas green tea drank even at a little amount can provide significant protection to postmenopausal women. Long-term consumption of tea was suggested by the specialists for a better result [35].

This review highlights that green tea intake on a regular basis has a remarkable role in lung cancer prevention in women. It was also shown that cigarette smokers who drank decaffeinated green tea regularly possess a limited risk compared to those smokers drank black tea. According to this review, it is very unfortunate that EGCG found in green tea may obstruct the medicinal effects of Bortezomib. Patients taking this medicine are suggested to avoid green tea [32].

Most of the studies performed to show the association between green tea and colorectal cancer provided us equivocal results. Among the studies which was considered while performing this study revealed that green tea has better effectiveness in women as men show an unhealthy lifestyle. Therefore, as a huge difference in the morbidity and pathogenesis of colorectal cancer with green tea consumption still exists between men and women [86], a further gender-specific study is suggested to have a clearer concept.

The information found from observational studies clarifies that green tea polyphenols as well as flavonoids have positive effects on prostate carcinogenesis, tumor cell growth inhibition, and therefore treatment of cancer [56]. How much prevention from prostate cancer is possible by drinking regular green tea still is not totally clear to us. For this, the more human clinical trials will be performed, the clearer the effects of green tea against prostate cancer will be.

Women drinking green tea at an excessive amount per day show better result against stomach cancer. Unfortunately, men due to their uncontrolled lifestyle, may suffer more from this type of cancer. The ideology of specialists is like that if men as well as women can regulate their lives in a proper way, the rate of being affected by stomach cancer will decrease.

Green tea remains neutral in preventing liver cancer in men. Women who drink green tea daily at a greater amount possess lesser risk to liver cancer. As tea consumption cannot protect men from this type of cancer, proper monitoring, proper diet and regular consultation with doctors may save them [44].

Caffeinated green tea may decrease the possibility of being affected by skin cancer. To protect the skin from excessive UV light exposure, washing the skin with green tea extract may help us [31]. Commercial production of face packs made of green tea extracts may be effective in preventing skin cancer at present.

Women drinking huge amounts of green tea are affected lesser by pancreatic cancer than those who drink tea at a lesser amount or never drink tea [64]. Green tea cannot show a better result in preventing men from this type of cancer. The dosage of green tea that may save women from pancreatic cancer is still unclear. Therefore, more clinical tests should be conducted to become sure of it.

Green tea polyphenols remain neutral in preventing men and women i.e., people of both sexes drinking green tea regularly show reduced risk in oral cancer. Both EGCG and ECG play their effective roles in inhibiting cancer cell growth. It has already been highlighted in this article that green tea extract having EGCG and ECG in it may strengthen dental bones and pulps. So, making toothpaste with green tea extract and selling it commercially may help us decrease the increasing number of oral cancer cases [66].

Caffeinated green tea consumption at a moderate amount may have positive effects on ovarian cancer prevention in women than those who drink decaffeinated green tea. Drinking green tea along with black tea combines EGCG with theaflavin which is a significantly useful combination against this type of cancer prevention.

As unhealthy diet and an unplanned and undisciplined lifestyle of people are mainly responsible for increasing the number of bladder cancer cases, most of the studies found a neutral relationship between green tea intake and bladder cancer reduction. However, drinking tea may reduce the risk in those than the non-drinkers. ECG and EGCG can both play vital roles in this cancer prevention [71]. The combination of black tea and green tea simultaneously may protect against bladder cancer.

There remains a strong contradiction about the influences of green tea against esophageal cancer. Green tea has no positive effect in smokers but in non-smokers it may partially help the patients survive this cancer [47]. As temperature is one of the major factors in this case, whether drinking tea at a hot temperature is better or not is still inconclusive. For clearer results, more clinical trials are suggested.

Though it is proven that green tea is much more effective against cancer as a natural agent, there are also a few limitations of this study. The mentionable limitations found are:

- As green tea is more popular in Asian countries, especially China, Japan, etc., tea consumption is higher in those areas. As a result, most of the epidemiological studies were performed on the people living in that region. As environment is a crucial factor in creating cancer types, epidemiological studies should be performed worldwide to obtain more concrete results [64].
- In a dose-manner way, how much green tea intake could show sufficient efficacy against cancer is still unknown. Studies with a wide range of the population drinking various amounts of tea should be examined and analyzed [51].
- Only observational studies cannot provide proper information about the association between green tea and cancer prevention. Population-based clinical trials should be conducted to be certain about the anti-cancer activity of green tea [54].
- It has been reported that green tea can prevent different types of cancer or can be used as treatment for a while by treating naturally without any side effects. However, how efficacious green tea would be against different sub cancer types is still unknown. Thus, further analysis with various sub-types of cancer could be performed [18].
- It has been reported in a number of literatures that drinking hot green tea may stimulate cancerous cell growth (i.e., stomach or esophageal cancer). Thus, population-based tests with tea at both hot and moderate temperatures should be performed to reach a result [35].
- Several harmful effects on the human body have been reported due to excessive green tea consumption owing to some of its structural components [9].

Several adverse effects of green tea are as follows: [9]

✓ Causing cytotoxicity of liver cells by EGCG
✓ Occurrence of oxidative DNA damage in pancreas and liver
✓ Causing frequent hyperglycemia in diabetic patients
✓ May cause renal failure by offering excessive aluminum to patients
✓ Its caffeine content may be malignant for heart disease patients

Thus, considering the above-mentioned harmful effects of green tea, awareness should be raised while drinking this beverage to find ways of relieving these effects. Studies focusing on these must be performed in future.

## 6. Conclusions and the Following Potentials

The findings of this review suggests that green tea polyphenols influence several signaling and metabolic pathways which result in inhibition of growth of tumor cell receptors, induction of cell apoptosis, proliferation and the occurrence of cell cycle arrest. That is why green tea polyphenols are considered effective against the fatal disease “cancer” due to their powerful antioxidant characteristics. EGCG plays the best role in cancer prevention or treatment in many cases. EGCG also acts on changing cancerous cell signaling pathways to inhibit their harmful effects on the body. EGCG stops abnormal cell growth, occur cell death in need to resist the cells to spread and thus becomes an essential agent against cancer. However, according to many studies, EGCG is very low bioavailable when it is taken as a supplement, or it can interfere with other intestinally absorbed substances and this interference may result in some difficulties in the human body. To elucidate the mechanism of EGCG, bioavailability of EGCG and efficacy of EGCG as a supplement might be the main research directions in the future.

## Figures and Tables

**Table 1:** Major beneficial constituents of green tea [9,10].

Name of the Constituents	Quantity (%)
Protein	15–20
Amino acids	1–4
Carbohydrates	5–7
Essential trace elements	5
Catechins	20–30
Lignin	7
Organic acid	2
Chlorophyll	0.5

**Table 3:** Epidemiological studies of the relationship between green tea consumption and human cancer risk.

Organ Sites	Green Tea Consumption Level	Country of the Study Conducted	Gender	Population Size	Results Found	References
Breast	≥5 cups/day ≤4 cups/day	Japan	Men and women	8552	High consumption level showed lower risk	[36]
Lung	n/a	China	Women	675	Consumption of green tea associated with reduced lung cancer risk	[32,49]
Colon-rectum	>5 cups/day	China	Men and women	>60,000	Insignificant increase in risk of colon cancer in regular drinkers than non-drinkers.	[47]
Prostate	≥5 cups/day ≤1 cup/day	Japan	Men	49,920	Green tea consumption may be associated with a reduced risk of prostate cancer	[47,82]
Stomach	≥5 cups/day ≥7 cups/day	Japan	Men	39,290	Greater consumption showed better result	[83]
Liver	≥5 cups/day 1 cup/day	Japan	Men and women	21,128	Greater intake possessed lesser risk	[44]
Skin	n/a				No dose-related result was found	
Pancreas	≥7 cups/day <1 cup/day	Japan	Men and women	100,000	No association between green tea consumption and cancer risk was found	[3]
Oral	≥5 cups/day ≤1 cup/day	Japan	Men and women	50,221	An inverse relation between green tea consumption and oral cancer	[47,84]
Ovary	≥4 cups/day	Australia	Men and women	2784	Significant reduction in ovary cancer risk with green tea intake	[69]
Bladder	≥5 cups/day	Canada	Men and women	3045	associated with green tea consumption.	[3]
Esophagus	≥300 g/month	China	Men and women	1884	Moderate level of consumption may reduce the risk but drinking tea > 65 °C has association with increased cancer risk	[81]

## Data Availability

The data presented in this study are available upon request from the corresponding author.
